# Gestational weight gain and perinatal outcomes in twin pregnancies: evidence-based insights from a Chinese population cohort

**DOI:** 10.1080/07853890.2026.2659391

**Published:** 2026-04-27

**Authors:** Jie Chen, Xu Cao, Yu Zhang, Yiting Li, Kangxin Wang, Xiayang Li, Yishuai Huang, Fuyang Zhao, Siyi Ren, Doudou Zhao, Wei Liu, Liqiong Guo, Pengfei Qu

**Affiliations:** ^a^ Translational Medicine Center, Northwest Women’s and Children’s Hospital, Xi’an, China; ^b^ Department of Epidemiology and Biostatistics, School of Public Health, Xi’an Jiaotong University Health Science Center, Xi’an, China; ^c^ Graduate Department, Xi’an Medical University, Xi’an, China; ^d^ School of Nursing, Shaanxi University of Chinese Medicine, Xianyang, China; ^e^ School of Disaster and Emergency Medicine, Tianjin University, Tianjin, China

**Keywords:** Body mass index, gestational weight gain, perinatal outcomes, twin pregnancy

## Abstract

**Background:**

Twin pregnancies carry perinatal risks. Gestational weight gain (GWG) is modifiable, but Institute of Medicine (IOM) twin recommendations may not suit Chinese women.

**Method:**

A retrospective cohort study included 3109 twin pregnancies delivered at Northwest Women’s and Children’s Hospital in Xi’an, China, during 2018–2022. Pre-pregnancy BMI was classified according to WHO criteria, with Chinese criteria used in sensitivity analyses. Optimal BMI-specific GWG ranges were defined as the interquartile range among low-risk pregnancies. GWG adequacy (below/within/above) was evaluated against study-derived and IOM-recommended ranges. Associations were estimated using multivariable generalized estimating equations, with threshold-effect and smooth-curve analyses to assess nonlinearity.

**Results:**

Optimal GWG ranges were: underweight, 15.91–23.68 kg; normal, 14.80–21.46 kg; overweight, 12.95–19.98 kg; obese, 7.03–17.76 kg. GWG below range increased risks of moderate preterm birth (MPTB) (OR 1.62, 95% CI 1.29–2.04), very preterm birth (VPTB) (OR 1.90, 95% CI 1.34–2.70), low birth weight (LBW) (OR 1.87, 95% CI 1.61–2.17), and small for gestational age (SGA) (OR 1.31, 95% CI 1.08, 1.59). Excessive GWG raised large for gestational age (LGA) risk (OR 1.98, 95% CI 1.59, 2.46) but lowered LBW (OR 0.69, 95% CI 0.59–0.81) and SGA (OR 0.75, 95% CI 0.60, 0.95). Nonlinear analyses showed U-shaped risks for MPTB, VPTB, and Apgar ≤ 7; inverted U-shaped for gestational age and birth weight; J-shaped for LGA.

**Conclusions:**

BMI-specific GWG recommendations derived from this large Chinese twin cohort are lower than IOM values. Both inadequate and excessive GWG increase adverse perinatal risks.

## 1. Introduction

With the increasing use of assisted reproductive technologies(ART), the global incidence of twin pregnancies has risen steadily in recent years. According to the WHO, the global twin birth rate increased from 9.1‰ in 1980 to 12‰ in 2015 [1]. Compared to singleton pregnancies, twin pregnancies are associated with substantially higher risks of maternal complications, including gestational hypertension and gestational diabetes, as well as adverse neonatal outcomes such as stillbirth, severe asphyxia, low birth weight (LBW) [2–4]. Although twin pregnancies account for a small proportion of all pregnancies, they contribute to approximately one-quarter of all low birth weight infants and nearly half of preterm births [5].

Gestational weight gain (GWG) serves as a vital measure of maternal nutritional status during pregnancy [6], and both inadequate and excessive gain increase the risk of adverse birth outcomes [7,8]. In singleton pregnancies, excessive GWG is associated with increased risks of macrosomia, cesarean delivery, and postpartum weight retention, while inadequate GWG is linked to intrauterine growth restriction, SGA, and preterm birth [9,10]. Similar associations have been observed in twin pregnancies: inadequate GWG increases the risk of PTB and fetal growth restriction, whereas excessive GWG raises the risk of gestational complications such as hypertensive disorders [11–13]. In 2009, the Institute of Medicine (IOM) proposed provisional GWG recommendations for twin pregnancies: 16.8–24.5 kg for women with normal pre-pregnancy BMI, 14.1–22.7 kg for those who are overweight, and 11.3–19.1 kg for women with obesity [14]. However, these guidelines were based primarily on Western populations and were designated by the IOM as interim recommendations due to the limited availability of high-quality evidence. Given the generally lower average BMI among Chinese women, along with their different lifestyle and ethnic characteristics, the applicability of the IOM recommendations to the Chinese population requires further investigation. Therefore, this study aimed to determine optimal GWG ranges stratified by BMI among Chinese women with twin pregnancies and to evaluate the associations between GWG and adverse perinatal outcomes, using a large retrospective cohort from Northwest Women and Children’s Hospital in Xi’an, China.

## 2. Materials and methods

### 2.1. Study design

This study was a single-center retrospective cohort study conducted at Northwest Women and Children’s Hospital in Xi’an, China. The study population consisted of women with twin pregnancies who were hospitalized and delivered at the hospital between January 2018 and December 2022. Study variables were retrieved from the hospital’s electronic inpatient record system.

### 2.2. Eligibility criteria (inclusion and exclusion)

**Figure 1.**
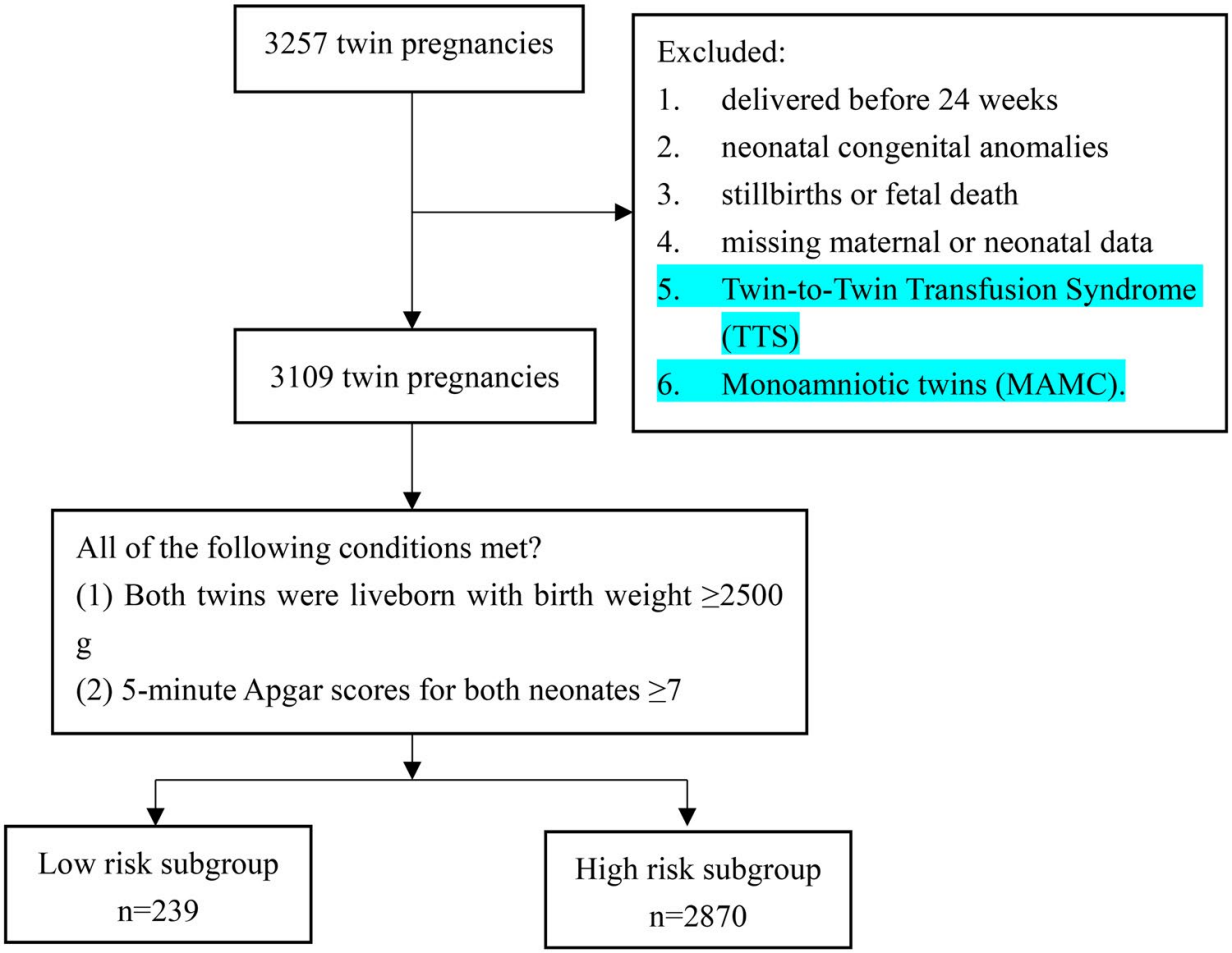
Flow diagram of study participants.

The exclusion criteria were as follows: (1) gestational age at delivery < 24 weeks; (2) stillbirth or fetal death; (3) twin pregnancies with fetal malformations; and (4) missing data of height, weight, or covariates; (5) Twin-to-Twin Transfusion Syndrome (TTS); (6) Monoamniotic twins (MAMC). A total of 3109 women met the inclusion criteria and were included in the final analysis (Figure 1).

### 2.3. Human ethics

Ethical approval was granted by the Northwest Women’s and Children’s Hospital Human Research Ethics Committee (No.202504301), and the requirement for written informed consent was waived given the study’s retrospective, observational design.

### 2.4. Exposure

The primary exposure in this study was GWG. Pre-pregnancy weight was self-reported at the first antenatal visit (8–12 weeks of gestation), while both height and weight were measured by trained nurses and re-measured before delivery. GWG was calculated as the difference between the body weight at delivery and the pre-pregnancy weight. Additionally, the GWG rate (kg/week) was calculated by dividing total GWG by gestational age at delivery (in weeks). This rate was compared to the IOM recommended weekly GWG for twin pregnancies, calculated by dividing the IOM-recommended total GWG at 37 weeks by 37 [15]. Based on this, each participant’s GWG was classified as below, within, or above the IOM-recommended range.

Pre-pregnancy BMI was calculated as weight in kilograms divided by the square of height in meters (kg/m^2^). BMI was categorized according to both WHO and Chinese criteria. The WHO classification is as follows: underweight, BMI < 18.5 kg/m^2^; normal weight, BMI 18.5–24.9 kg/m^2^; overweight, BMI 25.0–29.9 kg/m^2^; and obesity, BMI ≥ 30.0 kg/m^2^. The Chinese BMI classification system: underweight (BMI <18.5 kg/m^2^), normal weight (18.5–23.9 kg/m^2^), overweight (24.0–27.9 kg/m^2^), and obesity (≥28.0 kg/m^2^).

### 2.5. Study outcomes and definitions

The primary outcome of this study was a composite perinatal outcome, which was categorized into low-risk and high-risk subgroups. The low-risk subgroup included only those meeting all of the following criteria: (1) both twins were liveborn with birth weight ≥2500 g, (2) 5-minute Apgar scores for both neonates ≥7, and (3) gestational age at delivery ≥37 weeks. All other cases were classified as the high-risk subgroup. Other major outcomes are defined as follows:: Neonatal weight classifications included low birth weight (LBW; <2500 g measured within the first hour after birth), very low birth weight (VLBW; <1500 g), large for gestational age (LGA; birth weight exceeding the sex-specific 90th percentile for gestational age based on internationally recommended standards), and small for gestational age (SGA; birth weight below the sex-stratified 10th percentile for corresponding gestational age). Small vulnerable newborns (SVN) were defined as infants born preterm, SGA, or LBW, and were further subclassified into three subgroups: preterm non-SGA, term SGA, and preterm SGA [16].

### 2.6. Determination of optimal gestational weight gain

To determine the optimal range of GWG, we analyzed the distribution of GWG among women in the low-risk composite outcome subgroup. The optimal GWG range was defined as the interquartile range (25th to 75th percentile) of total GWG within this low-risk population.

### 2.7. Statistical analysis

Categorical variables are described using frequencies and percentages, with inter-group comparisons conducted *via* Chi-square or Fisher’s exact tests, as indicated. Continuous data are reported as mean ± standard deviation (x ± SD).and differences were assessed using one-way analysis of variance (ANOVA). Logistic regression analysis was used to examine the association between GWG and maternal outcomes, while generalized estimating equations (GEE) were employed to assess the relationship between categories of gestational weight gain and neonatal outcomes.

Odds ratios (ORs) and 95% confidence intervals (CIs) were estimated to quantify the associations between gestational weight gain (GWG) rate and adverse perinatal outcomes. To examine potential nonlinear relationships, threshold-effect models and smooth-curve fitting techniques were applied.

Threshold effects were assessed using a piecewise (two-stage) logistic regression model. An iterative procedure was conducted to identify potential inflection points across the observed range of GWG rate (kg/week). For each candidate threshold, a segmented regression model was fitted, and the optimal inflection point was determined by maximizing model fit, defined as the minimum residual sum of squares. The statistical significance of the identified threshold was evaluated using the likelihood ratio test by comparing the segmented model with a conventional single-line (linear) model.

In addition, restricted cubic spline (RCS) regression was employed to flexibly model the continuous nonlinear association between GWG rate and pregnancy outcomes. Nonlinearity was assessed by comparing the goodness-of-fit between linear and spline-based models using the likelihood ratio test. A *p* value < 0.05 was considered indicative of a statistically significant nonlinear relationship, supporting the presence of potential inflection points.

All models were adjusted for potential confounders based on previous literature, including maternal age, pre-pregnancy BMI, cesarean section history, polycystic ovary syndrome, education level, parity, adverse pregnancy history, mode of conception, chorionicity, hypertensive disorders, gravidity, and diabetes.

To validate the robustness of our findings, we conducted a sensitivity analysis by re-analyzing the data using the Chinese BMI classification standards.

All statistical analyses were performed using R software (http://www.r-project.org). A two-sided *p* value <0.05 was considered statistically significant.

## 3. Results

### 3.1. Baseline characteristics by BMI category

A total of 3109 mother–twin pairs were included in this study, according to the WHO BMI classification criteria, including 364 in the underweight group, 2204 in the normal weight group, 461 in the overweight group, and 80 in the obese group. Regarding demographic characteristics, the underweight group had the highest GWG rate (0.51 ± 0.14 kg). The normal weight group had the highest proportion of women with university-level education or above (52.22%), while the overweight group had the highest average maternal age (31.03 ± 3.80 years), and the obese group had the youngest average maternal age (29.77 ± 3.05 years), the lowest proportion with higher education (33.75%), the highest incidence of PCOS. (13.75%), and the lowest GWG rate (0.36 ± 0.17 kg/week).

In terms of perinatal outcomes, the underweight group had the lowest cesarean section rate (96.15%) and the highest rates of LBW (67.86%) and SGA (16.7%). The overweight group had the highest rates of MPTB (22.13%) and VPTB (9.33%), and also had the highest proportion of newborns with 1-minute (7.05%) and 5-minute Apgar scores ≤7 (1.84%). The highest incidence of LGA was observed in the obese group (13.75%) (Table 1).

**Table 1.** Baseline characteristics and outcomes of study population by BMI groups using WHO criteria.

Characteristics or outcomes	Underweight *n* = 364	Normal weight *n* = 2204	Overweight *n* = 461	Obese *n* = 80	*p* value
Maternal age (year)	29.89 ± 3.87	30.82 ± 3.66	31.03 ± 3.80	29.77 ± 3.05	<0.001
<35 year	318 (87.36)	1872 (84.94)	375 (81.34%)	78 (97.50%)	0.001
≥35 year	46 (12.64%)	332 (15.06%)	86 (18.66%)	2 (2.50%)	
Educational status, *n*(%)					<0.001
Junior high school and below	83 (22.80%)	486 (22.05%)	149 (32.32%)	34 (42.50%)	
Senior high school and Associate degree	101 (27.75%)	567 (25.73%)	148 (32.10%)	19 (23.75%)	
University and beyond	180 (49.45%)	1151 (52.22%)	164 (35.57%)	27 (33.75%)	
GWG ((kg/week)), mean ± SD	0.51 ± 0.14	0.48 ± 0.15	0.43 ± 0.17	0.36 ± 0.17	<0.001
Gravidity (*n*), mean ± SD	0.81 ± 1.05	0.81 ± 1.05	0.83 ± 1.10	0.97 ± 1.16	0.609
Parity (*n*), mean ± SD	0.24 ± 0.45	0.27 ± 0.49	0.23 ± 0.46	0.29 ± 0.56	0.197
Pregnancy loss history, *n* (%)	150 (41.21%)	825 (37.43%)	189 (41.00%)	34 (42.50%)	0.268
PCOS, *n* (%)	15 (4.12%)	118 (5.35%)	36 (7.81%)	11 (13.75%)	0.002
Pre-pregnancy diabetes, *n* (%)	2 (0.55%)	29 (1.32%)	18 (3.90%)	5 (6.25%)	<0.001
GDM, *n* (%)	56 (15.38%)	520 (23.59%)	174 (37.74%)	27 (33.75%)	<0.001
Pre-pregnancy hypertension, *n* (%)	2 (0.55%)	16 (0.73%)	13 (2.82%)	3 (3.75%)	<0.001
GHDs, *n* (%)	27 (7.42%)	218 (9.89%)	72 (15.62%)	26 (32.50%)	<0.001
Past cesarean delivery, *n* (%)	35 (9.62%)	292 (13.25%)	58 (12.58%)	11 (13.75%)	0.285
Methods of conception, *n* (%)					<0.001
Spontaneous	213 (58.52%)	1057 (47.96%)	160 (34.71%)	25 (31.25%)	
Fertility-enhancing drugs for OI and/or IUI	7 (1.92%)	106 (4.81%)	23 (4.99%)	8 (10.00%)	
ART	144 (39.56%)	1041 (47.23%)	278 (60.30%)	47 (58.75%)	
Chorionicity, *n* (%)					0.008
Dichorionic	260 (71.43%)	1664 (75.50%)	362 (78.52%)	70 (87.50%)	
Monochorionic	104 (28.57%)	540 (24.50%)	99 (21.48%)	10 (12.50%)	
Cesarean delivery, *n* (%)	350 (96.15%)	2155 (97.78%)	445 (96.53%)	79 (98.75%)	0.140
Gestational age (week)	35.48 ± 1.78	35.59 ± 1.72	35.00 ± 2.16	35.39 ± 1.84	<0.001
PTB, *n* (%)	306 (84.07%)	1880 (85.30%)	412 (89.37%)	67 (83.75%)	0.093
MPTB, *n* (%)	63 (17.31%)	266 (12.07%)	102 (22.13%)	11 (13.75%)	<0.001
VPTB, *n* (%)	17 (4.67%)	102 (4.63%)	43 (9.33%)	6 (7.50%)	<0.001
Birth weight, mean ± SD	2262.70 ± 427.69	2358.80 ± 420.05	2311.45 ± 516.26	2376.47 ± 581.57	<0.001
LBW, *n* (%)	494 (67.86%)	2627 (59.60%)	539 (58.46%)	94 (58.75%)	<0.001
VLBW, *n* (%)	46 (6.32%)	184 (4.17%)	76 (8.24%)	12 (7.50%)	<0.001
SGA, *n* (%)	122 (16.76%)	468 (10.62%)	95 (10.30%)	19 (11.88%)	<0.001
LGA, *n* (%)	33 (4.53%)	343 (7.78%)	114 (12.36%)	22 (13.75%)	<0.001
SVN, *n* (%)	663 (91.07%)	3953 (89.68%)	841 (91.21%)	144 (90.00%)	0.403
Preterm non-SGA, *n* (%)	507 (69.64%)	3354 (76.09%)	735 (79.72%)	118 (73.75%)	<0.001
Term SGA, *n* (%)	12 (1.65%)	33 (0.75%)	3 (0.33%)	3 (1.88%)	<0.001
Preterm SGA, *n* (%)	105 (14.42%)	406 (9.21%)	89 (9.65%)	16 (10.00%)	0.002
Apagar1, *n* (%)					<0.001
>7	698 (95.88%)	4268 (96.82%)	857 (92.95%)	152 (95.00%)	
≤7	30 (4.12%)	140 (3.18%)	65 (7.05%)	8 (5.00%)	
Apagar5, *n* (%)					<0.001
>7	723 (99.31%)	4383 (99.43%)	905 (98.16%)	158 (98.75%)	
≤7	5 (0.69%)	25 (0.57%)	17 (1.84%)	2 (1.25%)	

SD: standard deviation; GWG: gestational weight gain; PCOS: Polycystic Ovary Syndrome; GDM: gestational diabetes mellitus; GHDs: gestational hypertensive disorders; OI: ovulation induction; IUI: intrauterine insemination; ART: Assisted reproductive technology; PTB: preterm birth; MPTB: moderate preterm birth; VPTB: very preterm birth; LBW: low birth weight; VLBW: very low birth weight; SGA: small for gestational age; LGA: large for gestational age; SVN: small vulnerable newborns.

### 3.2. Optimal GWG ranges

**Figure 2.**
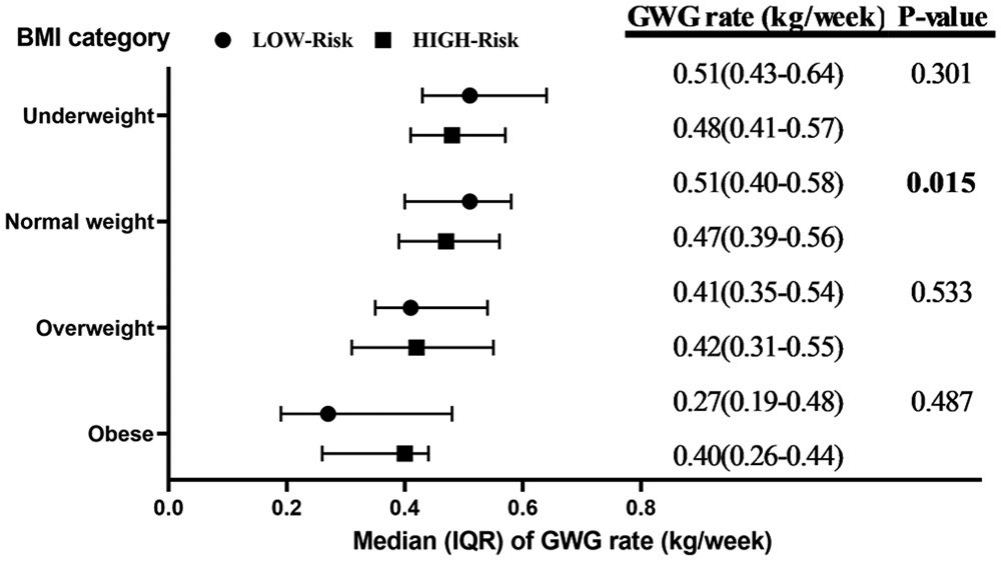
Distribution of GWG rate in the low- and high-risk subgroups. Note: In the low-risk group, there were 22 individuals in the underweight category, 173 in the normal weight category, 37 in the overweight category, and 7 in the obese category.

To determine the optimal GWG range, we estimated the distribution of overall GWG rate in both the low-risk and high-risk subgroups (Figure 2). We found that, in the underweight and normal weight groups, the median GWG rate in the low-risk subgroup was higher than that in the high-risk subgroup, while in the obese group, the median GWG rate in the low-risk subgroup was lower than in the high-risk group. Based on the above GWG distributions, we further determined the total GWG recommendations for each BMI group (Table 2): Underweight group: total GWG 15.91–23.68 kg, corresponding to a weekly GWG rate of 0.43–0.64 kg/wk, Normal weight group: total GWG 14.80–21.46 kg, weekly GWG rate of 0.40–0.58 kg/wk, Overweight group: total GWG 12.95–19.98 kg, weekly GWG rate of 0.35–0.54 kg/wk, Obese group (≥28 kg/m^2^): total GWG 7.03–17.76 kg, weekly GWG rate of 0.19–0.48 kg/wk.

**Table 2.** IOM and our study recommendations for gestational weight gain in twin gestations.

	IOM recommended		Our study recommended	
Pre-pregnancy BMI	Total weight gain at 37 wk in kg (lb)	Weekly weight gain kg/wk (lb/wk)	Total weight gain at 37 wk in kg (lb)	Weekly weight gain kg/wk (lb/wk)
Underweight (BMI < 18.5 kg/m^2^)	/	/	15.91–23.68 (35.15–52.17)	0.43–0.64 (0.95–1.41)
Normal weight (18.5–24.9 kg/m^2^)	16.8–24.5 (37.0–54.0)	0.45–0.66 (1.03–1.50)	14.80–21.46 (32.56–47.36)	0.40-0.58 (0.88–1.28)
Overweight (25–29.9 kg/m^2^)	14.1–22.7 (31.1–50.0)	0.38–0.61 (0.86–1.39)	12.95–19.98 (28.49–44.03)	0.35–0.54 (0.77–1.19)
Obese (≥30 kg/m^2^)	11.3–19.1 (24.9–42.1)	0.31–0.51 (0.69–1.17)	7.03–17.76 (15.54–39.22)	0.19–0.48 (0.42–1.06)

*Note:* The study-recommended GWG ranges are based on the interquartile range (25th–75th percentiles) of GWG in the low-risk group and stratified by WHO BMI categories.lb is the abbreviation of pounds. wk is short for week.

### 3.3. Association between GWG and perinatal outcomes

After adjusting for confounding factors, GEE results showed that GWG below the study-recommended range significantly increased the risk of MPTB (OR = 1.62, 95% CI: 1.29–2.04), VPTB (OR = 1.90, 95% CI: 1.34–2.70), LBW(OR = 1.87, 95% CI: 1.61–2.17), VLBW (OR = 1.99, 95% CI: 1.45–2.74), SGA (OR = 1.31, 95% CI: 1.08, 1.59), 1-minute Apgar score ≤7 (OR = 2.16, 95% CI: 1.47–3.17), 5-minute Apgar score ≤7 (OR = 3.33, 95% CI: 1.39–7.98), GWG above the study-recommended range was significantly associated with increased risk of LGA (OR = 1.98, 95% CI: 1.59, 2.46), and reduced risk of LBW (OR = 0.69, 95% CI: 0.59–0.81) and SGA (OR =0.75, 95% CI:0.60, 0.95). Above or below IOM recommendations are generally consistent with the above results (Table 3).

**Table 3.** Association of gestational weight gain categories based on IOM and the study recommendations with perinatal outcomes.

	GWG rate below IOM	GWG rate above IOM	GWG rate below the study	GWG rate above the study
Perinatal outcomes	*β*/OR(95% CI)	*β*/OR(95% CI)	*β*/OR(95% CI)	*β*/OR(95% CI)
Cesarean delivery	0.55 (0.32, 0.94)*	0.93 (0.37, 2.34)	0.65 (0.40, 1.07)	1.28 (0.66, 2.51)
Gestational age (week)	−0.30 (−0.44, −0.16)**	0.07 (−0.14, 0.29)	−0.37 (−0.51, −0.22)**	0.11 (−0.05, 0.27)
PTB	1.07 (0.82, 1.39)	0.72 (0.49, 1.06)	0.84 (0.65, 1.08)	0.82 (0.61, 1.10)
MPTB	1.45 (1.14, 1.84)**	0.91 (0.61, 1.34)	1.62 (1.29, 2.04)**	0.85 (0.64, 1.13)
VPTB	1.66 (1.16, 2.38)**	0.94 (0.51, 1.74)	1.90 (1.34, 2.70)**	0.68 (0.42, 1.10)
Birth weight (g)	−129.12 (−160.22, −98.03)**	85.91 (39.45, 132.36)**	−151.80 (−183.72, −119.88)**	87.34 (51.99, 122.68)**
LBW	1.64 (1.42, 1.89)**	0.67 (0.54, 0.83)**	1.87 (1.61, 2.17)**	0.69 (0.59, 0.81)**
VLBW	1.82 (1.31, 2.53)**	0.67 (0.37, 1.21)	1.99 (1.45, 2.74)**	0.70 (0.46, 1.08)
SGA	1.35 (1.12, 1.64)**	0.77 (0.61, 0.98)**	1.31 (1.08, 1.59)**	0.75 (0.60, 0.95)*
LGA	0.50 (0.38, 0.66)**	1.69 (1.58, 2.43)**	0.50 (0.38, 0.66)**	1.98 (1.59, 2.46)**
SVN	1.0 (0.7, 1.3)	0.8 (0.6, 1.1)	1.0 (0.8, 1.3)	0.9 (0.6, 1.2)
Preterm non-SGA	0.75 (0.63, 0.90)**	1.07 (0.88, 1.31)*	0.78 (0.65, 0.93)**	1.13 (0.93, 1.38)
Term SGA	2.08 (0.98, 4.42)	1.46 (0.65, 3.31)	2.44 (1.17, 5.07)*	1.65 (0.76, 3.57)
Preterm SGA	1.30 (1.06, 1.60)*	0.72 (0.55, 0.93)*	1.24 (1.00, 1.52)*	0.68 (0.53, 0.87)*
Apagar1 ≤ 7	1.92 (1.28, 2.87)**	1.01 (0.52, 1.97)	2.16 (1.47, 3.17)**	0.93 (0.57, 1.54)
Apagar5 ≤ 7	2.93 (1.10, 7.79)*	3.59 (1.24, 10.39)*	3.33 (1.39, 7.98)**	2.40 (0.96, 6.00)

*Note:* The study-recommended GWG ranges are based on the interquartile range (25th–75th percentiles) of GWG in the low-risk group and stratified by WHO BMI categories. SD: standard deviation; PTB: preterm birth; MPTB: moderate preterm birth; VPTB: very preterm birth; LBW: low birth weight; VLBW: very low birth weight; SGA: small for gestational age; LGA: large for gestational age; SVN: small vulnerable newborns. Adjust model adjust for: maternal age, educational status, gravidity, parity, pregnancy loss history, PCOS (Polycystic Ovary Syndrome), pre-pregnancy diabetes, GDM (gestational diabetes mellitus), pre-pregnancy hypertension, GHDs (gestational hypertensive disorders), Past cesarean delivery, Methods of conception, Chorionicity, BMI (body mass index). *p < 0.05, **p < 0.01.

### 3.4. Stratified analysis results

In the underweight group (Table 4), GWG below the study-recommended range was associated with increased risks of LBW, SGA, Preterm non-SGA, and Preterm SGA, and with decreased birth weight and LGA. GWG above study-recommended recommended range was associated with increased birth weight and increased risk of LGA, and also with reduced risk of MPTB and LBW.

**Table 4.** Association of gestational weight gain categories based on our study recommendations with perinatal outcomes in underweight group.

	GWG rate below the study		GWG rate above the study	
Perinatal Outcomes	*β*/OR(95% CI)	*p* value	*β*/OR(95% CI)	*p* value
Cesarean delivery	1.20 (0.34, 4.28)	0.780	/	
Gestational age (week)	0.02 (−0.37, 0.41)	0.928	0.60 (0.07, 1.13)	0.026
PTB	0.69 (0.36, 1.34)	0.274	0.55 (0.25, 1.23)	0.144
MPTB	1.06 (0.56, 2.00)	0.857	0.32 (0.10, 0.98)	0.047
VPTB	1.33 (0.46, 3.83)	0.596	/	
Birth weight (g)	−125.56 (−207.97, −43.15)	0.003	162.07 (72.77, 251.38)	<0.001
LBW	1.90 (1.21, 2.97)	0.005	0.56 (0.33, 0.94)	0.029
VLBW	0.88 (0.37, 2.07)	0.767	/	
SGA	1.95 (1.20, 3.18)	0.007	0.91 (0.46, 1.80)	0.792
LGA	0.12 (0.02, 0.72)	0.020	2.65 (1.15, 6.12)	0.023
SVN	1.0 (0.5, 2.2)	0.967	0.6 (0.2, 1.4)	0.194
Preterm non-SGA	0.52 (0.33, 0.82)	0.006	0.70 (0.38, 1.31)	0.268
Term SGA	5.48 (0.96, 31.37)	0.056	1.76 (0.24, 13.08)	0.578
Preterm SGA	1.78 (1.05, 3.00)	0.032	0.80 (0.36, 1.77)	0.585

*Note:* Underweight is classified according to WHO BMI categories; SD: standard deviation; PTB: preterm birth; MPTB: moderate preterm birth; VPTB: very preterm birth; LBW: low birth weight; VLBW: very low birth weight; SGA: small for gestational age; LGA: large for gestational age; SVN: small vulnerable newborns.

Adjust model adjust for: maternal age, educational status, gravidity, parity, pregnancy loss history, PCOS (Polycystic Ovary Syndrome), pre-pregnancy diabetes, GDM (gestational diabetes mellitus), pre-pregnancy hypertension, GHDs (gestational hypertensive disorders), Past cesarean delivery, Methods of conception, Chorionicity.

In the normal weight group, GWG below the study-recommended range was associated with increased risks of shortened gestational age, moderate and VPTB, reduced birth weight, LBW, VLBW, SGA, and 1-minute Apgar ≤ 7, and with decreased risk of preterm non-SGA, LGA and Cesarean delivery. GWG above the recommended range was associated with increased risk of LGA and 5-minute Apgar ≤ 7, and with decreased risk of LBW, Preterm SGA.

In the overweight group, GWG below the study-recommended range was associated with increased risk of LBW, VLBW, SGA, preterm SGA, and 1-minute Apgar ≤ 7, 5-minute Apgar ≤ 7. GWG above the study-recommended range was associated with increased risks of birth weight, LGA, and reduced risk of Gestational age, MPTB, LBW.

In the Obese group, GWG above the study-recommended range was associated with increased risks of gestational age, birth weight. The group above the IOM recommended range was associated with decreased risk of LBW (Table 5).

**Table 5.** Association of gestational weight gain categories based on the IOM and study recommendations with perinatal outcomes stratified by WHO BMI categories.

		GWG rate below IOM	GWG rate above IOM	GWG rate below the study	GWG rate above the study
BMI categories	Perinatal outcomes	*β*/OR(95% CI)	*β*/OR(95% CI)	*β*/OR(95% CI)	*β*/OR(95% CI)
Normal weight	Cesarean delivery	0.53 (0.28, 1.00) *	0.83 (0.29, 2.32)	0.51 (0.27, 0.97) *	1.09 (0.47, 2.54)
	Gestational age (week)	−0.27 (−0.43, −0.12)**	0.05 (−0.18, 0.28)	−0.43 (−0.59, −0.27)**	−0.05 (−0.24, 0.13)
	PTB	1.11 (0.85, 1.45)	0.76 (0.52, 1.12)	0.75 (0.55, 1.02)	0.75 (0.55, 1.04)
	MPTB	1.41 (1.06, 1.87)*	0.88 (0.56, 1.39)	1.93 (1.42, 2.60)**	1.22 (0.86, 1.73)
	VPTB	1.89 (1.21, 2.97)*	1.08 (0.53, 2.20)	2.51 (1.59, 3.97)**	0.91 (0.51, 1.65)
	Birth weight (g)	−117.75 (−150.02, −85.48)**	92.80 (43.30, 142.31)**	−157.58 (−193.26, −121.89)**	55.26 (14.52, 95.99)**
	LBW	1.61 (1.37, 1.89)**	0.66 (0.52, 0.84)**	1.97 (1.66, 2.35)**	0.76 (0.63, 0.91)**
	VLBW	1.65 (1.12, 2.45)*	0.64 (0.31, 1.30) 0.2165	2.35 (1.54, 3.59)**	1.01 (0.59, 1.72)
	SGA	1.24 (0.98, 1.57)	0.75 (0.56, 1.01)	1.14 (0.90, 1.44)	0.74 (0.56, 0.97)*
	LGA	0.45 (0.32, 0.64)**	1.91 (1.47, 2.48)**	0.49 (0.34, 0.70)**	1.93 (1.49, 2.50)**
	SVN	0.9 (0.6, 1.2)	0.8 (0.6, 1.2)	0.9 (0.7, 1.3)	0.9 (0.6, 1.2)
	Preterm non-SGA	0.75 (0.61, 0.93)**	1.07 (0.84, 1.37)	0.80 (0.68, 0.94)**	1.14 (0.90, 1.45)
	Term SGA	2.14 (0.91, 5.01)	1.27 (0.43, 3.72)	1.91 (0.81, 4.53)	1.27 (0.47, 3.46)
	Preterm SGA	1.16 (0.90, 1.49)	0.70 (0.51, 0.95)*	1.06 (0.82, 1.37)	0.66 (0.49, 0.88)*
	Apagar1 ≤ 7	1.58 (0.97, 2.57)	0.94 (0.43, 2.05)	2.20 (1.31, 3.67)**	1.24 (0.67, 2.28)
	Apagar5 ≤ 7	0.99 (0.28, 3.53)	2.07 (0.63, 6.81)	3.17 (0.85, 11.77)	4.17 (1.31, 13.27)*
Overweight	Cesarean delivery	0.52 (0.17, 1.57)	1.68 (0.17, 16.26)	0.79 (0.23, 2.71)	1.10 (0.28, 4.27)
	Gestational age (week)	−0.51 (−0.93, −0.09)*	0.02 (−0.65, 0.70)	−0.37 (−0.83, 0.08)	0.53 (0.04, 1.01)*
	PTB	0.86 (0.44, 1.68)	0.73 (0.27, 2.01)	1.15 (0.55, 2.39)	1.03 (0.47, 2.27)
	MPTB	1.74 (1.07, 2.84)*	1.17 (0.51, 2.68)	1.35 (0.80, 2.26)	0.43 (0.22, 0.85)*
	VPTB	1.37 (0.70, 2.71)	0.85 (0.23, 3.08)	1.27 (0.62, 2.60)	0.47 (0.18, 1.25)
	Birth weight (g)	−205.05 (−296.62, −113.48)**	42.60 (−95.77, 180.97)	−168.65 (−270.32, −66.97)**	165.72 (62.41, 269.04)**
	LBW	2.05 (1.42, 2.95)**	0.66 (0.38, 1.15)	1.81 (1.20, 2.73)**	0.48 (0.32, 0.73)**
	VLBW	2.73 (1.46, 5.12)**	0.93 (0.30, 2.87)	2.22 (1.11, 4.44)*	0.61 (0.24, 1.55)
	SGA	1.96 (1.15, 3.34)*	0.94 (0.50, 1.79)	1.98 (1.16, 3.37)*	0.89 (0.47, 1.65)
	LGA	0.65 (0.38, 1.11)	1.60 (0.99, 2.61)	0.58 (0.33, 1.02)	1.68 (1.05, 2.70)
	SVN	2.0 (0.9, 4.4)	1.1 (0.5, 2.6)	1.7 (0.7, 4.1)	0.9 (0.4, 2.2)
	Preterm non-SGA	0.81 (0.50, 1.31)	1.14 (0.65, 1.98)	0.81 (0.50, 1.32)	1.14 (0.66, 1.96)
	Term SGA	/	4.85 (0.52, 45.04)	/	5.20 (0.45, 60.19)
	Preterm SGA	2.14 (1.24, 3.70)**	0.92 (0.46, 1.80)	2.09 (1.21, 3.61)**	0.85 (0.44, 1.64)
	Apagar1 ≤ 7	3.03 (1.40, 6.57)**	1.55 (0.43, 5.59)	2.90 (1.28, 6.57)*	0.92 (0.33, 2.59)
	Apagar5 ≤ 7	28.93 (4.70, 178.17)**	31.19 (2.83, 344.11)**	19.57 (1.72, 222.99)*	9.27 (0.71, 120.57)
Obese	Cesarean delivery	/	/	/	/
	Gestational age (week)	−0.55 (−1.64, 0.54)	1.04 (−0.30, 2.37)	0.18 (−1.09, 1.45)	1.25 (0.03, 2.48)*
	PTB	0.61 (0.08, 4.40)	0.57 (0.04, 9.15)	0.57 (0.04, 8.01)	0.36 (0.04, 3.40)
	MPTB	4.57 (0.36, 57.96)	0.25 (0.01, 9.90)	2.63 (0.27, 25.58)	0.16 (0.00, 5.69)
	VPTB	3.79 (0.25, 57.90)	/	0.49 (0.03, 8.93)	/
	Birth weight (g)	−49.57 (−287.85, 188.70)	399.56 (137.03, 662.08)**	132.23 (−193.24, 457.70)	496.55 (271.16, 721.93)**
	LBW	0.90 (0.24, 3.34)	0.14 (0.03, 0.63)*	0.37 (0.07, 1.90)	0.10 (0.02, 0.43)**
	VLBW	0.87 (0.04, 19.66)	/	0.79 (0.05, 11.96)	/
	SGA	0.25 (0.05, 1.32)	0.07 (0.01, 0.94)*	0.65 (0.14, 3.06)	0.07 (0.00, 1.22)
	LGA	2.73 (0.55, 13.64)	/	2.02 (0.36, 11.39)	/
	SVN	0.3 (0.0, 13.6)	0.2 (0.0, 2.7)	0.1 (0.0, 3.7)	/
	Preterm non-SGA	1.24 (0.13, 11.72)	3.99 (0.83, 19.30)	0.72 (0.18, 2.88)	7.57 (1.60, 35.71)*
	Term SGA	/	/	/	/
	Preterm SGA	0.26 (0.05, 1.49)	0.08 (0.01, 0.77)*	0.18 (0.02, 1.89)	0.06 (0.01, 0.57)*

*Note:* The study-recommended GWG ranges are based on the interquartile range (25th–75th percentiles) of GWG in the low-risk group and stratified by WHO BMI categories. SD: standard deviation; PTB: preterm birth; MPTB: moderate preterm birth; VPTB: very preterm birth; LBW: low birth weight; VLBW: very low birth weight; SGA: small for gestational age; LGA: large for gestational age; SVN: small vulnerable newborns. Adjust model adjust for: maternal age, educational status, gravidity, parity, pregnancy loss history, PCOS (Polycystic Ovary Syndrome), pre-pregnancy diabetes, GDM (gestational diabetes mellitus), pre-pregnancy hypertension, GHDs (gestational hypertensive disorders), Past cesarean delivery, Methods of conception, Chorionicity. *p < 0.05, **p < 0.01.

### 3.5. Threshold effects and smoothing curve analysis

**Figure 3.**
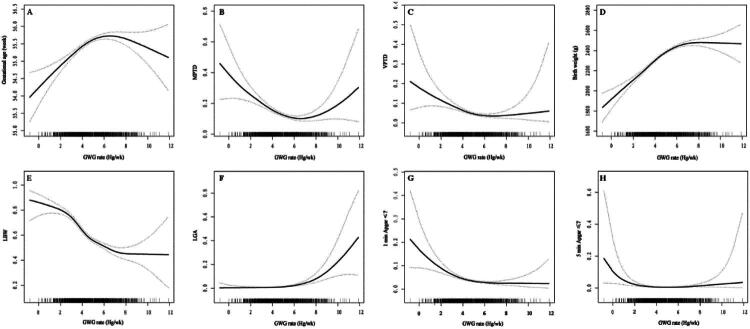
Smooth-fit plot illustrating the relationship between GWG rate and perinatal outcomes. MPTB: moderate preterm birth; VPTB: very preterm birth; LBW: low birth weight; LGA: large for gestational age; Models adjust for: maternal age, educational status, gravidity, parity, pregnancy loss history, PCOS (Polycystic Ovary Syndrome), pre-pregnancy diabetes, GDM (gestational diabetes mellitus), pre-pregnancy hypertension, GHDs (gestational hypertensive disorders), Past cesarean delivery, Methods of conception, Chorionicity, BMI (body mass index). Hg is the abbreviation of 100 grams. wk is short for week. (A) The smooth-fit plot of the relationship between GWG rate and gestational age; (B) The smooth-fit plot of the relationship between GWG rate and moderate preterm birth; (C) The smooth-fit plot of the relationship between GWG rate and very preterm birth; (D) The smooth-fit plot of the relationship between GWG rate and birth weight; E: The smooth-fit plot of the relationship between GWG rate and low birth weight; (F) The smooth-fit plot of the relationship between GWG rate and large for gestational age; (G) The smooth-fit plot of the relationship between GWG rate and 1 min Apagar ≤ 7; (H) The smooth-fit plot of the relationship between GWG rate and 5 min Apagar ≤ 7.

The risk of LBW decreased continuously with increasing GWG rate. MPTB and VPTB, as well as 1- and 5-minute Apgar scores ≤ 7, displayed U-shaped associations. Gestational age and birth weight showed inverted U-shaped patterns. The association between GWG and LGA followed a classic J-shaped curve; LGA risk was minimal at low GWG rates but increased sharply beyond a certain threshold (Figure 3). Threshold effect analysis identified the following critical points for GWG rate: 5.5 hg/wk for gestational age, birth weight, LBW. 4.2 hg/wk for 1-minute Apgar score, 5-minute Apgar score, VLBW, and SGA.6.5 hg/wk for MPTB, VPTB, and LGA (Table 6).

**Table 6.** Threshold effect analysis of gestational weight gain rate on perinatal outcomes.

Perinatal outcomes	One-line linear regression model	cutoff value (K)	<K (hg/week)	>K (hg/week)	Log-likelihood ratio test
Gestational age	0.14(0.09, 0.18)	5.5	0.27 (0.19, 0.34)	−0.09 (−0.18, 0.01)	<0.001
PTB	0.98 (0.92, 1.05)	3.8	0.86 (0.86, 0.86)	1.01 (0.92, 1.08)	0.2811
MPTB	0.86 (0.79, 0.93)	6.9	0.79 (0.73, 0.85)	1.68 (1.27, 2.21)	<0.001
VPTB	0.81 (0.70, 0.81)	6.0	0.70 (0.62, 0.79)	1.28 (0.96, 1.71)	<0.001
Birth weight	52.40 (42.49, 62.30)	5.5	82.93 (67.95, 97.91)	−0.90 (−22.79, 20.99)	<0.001
LBW	0.81 (0.77, 0.84)	5.4	0.71 (0.67, 0.77)	0.96 (0.88, 1.06)	<0.001
VLBW	0.79 (0.71, 0.88)	4.2	0.67 (0.56, 0.81)	0.90 (0.77, 1.05)	0.0436
SGA	0.21 (0.11, 0.40)	4.1	0.08 (0.02, 0.33)	0.35 (0.14, 0.85)	0.1366
SVN	0.89 (0.85, 1.06)	4.0	0.73 (0.64, 1.05)	0.86 (0.83, 1.07)	0.4030
LGA	21.22 (11.29, 39.90)	6.5	46.78 (18.21, 120.16)	2.56 (0.28, 23.61) 0.4065	0.0407
Apagar1 ≤ 7	0.83 (0.72, 0.95)	4.3	0.67 (0.54, 0.82)	1.00 (0.82, 1.21)	0.0169
Apagar5 ≤ 7	0.98 (0.70, 1.38)	4.3	0.47 (0.34, 0.64)	1.58 (1.23, 2.02)	<0.0001

SD: standard deviation; PTB: preterm birth; MPTB: moderate preterm birth; VPTB: very preterm birth; LBW: low birth weight; VLBW: very low birth weight; SGA: small for gestational age; LGA: large for gestational age; SVN: small vulnerable newborns. hg is the abbreviation for hectogram.

### 3.6. Sensitivity analysis

A sensitivity analysis was performed using the Chinese BMI classification system. Based on the Chinese BMI criteria, the recommended total and weekly GWG ranges were as follows: Underweight group: total GWG 15.91–23.68 kg, weekly GWG 0.43–0.64 kg, Normal weight group: total GWG 15.17–21.46 kg, weekly GWG 0.41–0.58 kg, Overweight group: total GWG 12.58–19.98 kg, weekly GWG 0.34–0.54 kg, Obese group: total GWG 9.62–18.50 kg, weekly GWG 0.26–0.50 kg (Figure S1 and Table S1)

GEE results showed that, in the normal weight group, inadequate GWG was significantly associated with an increased risk of LBW and decreased risks of LGA. While excessive GWG was consistently associated with an elevated risk of LGA. In the overweight group, above GWG was also associated with a persistent increase in the risk of LGA. The results of the sensitivity analysis were largely consistent with those of the main analysis (Table S2).

## 4. Discussion

### 4.1. Main findings

Based on a retrospective cohort of 3109 twin pregnancies in Northwest China, we proposed optimal GWG ranges for twin pregnancies in the Chinese population, specifically addressing the underweight group a category not covered in IOM guidelines. Overall, our recommended GWG ranges were lower than those in the IOM guidelines. This study further elucidated the association between inadequate or excessive GWG and adverse maternal-infant outcomes in twin pregnancies according to pre-pregnancy BMI categories. Our main findings are as follows: inadequate GWG was significantly associated with increased risks of MPTB, VPTB, LBW, and SGA; whereas excessive GWG was associated with increased risks of LGA, and decreased incidence of LBW and SGA.

Furthermore, while this association trend is generally consistent with IOM recommendations, our estimated optimal GWG range at 37 weeks appears more predictive of adverse outcomes than the IOM-recommended range: the effect of GWG on birth weight under the IOM model was weaker than in this study (*β* = −129.12 vs. *β* = −151.80 g for insufficient GWG; *β* = 85.91 g vs. *β* = 87.34 g for excessive GWG). Similarly, the LGA risk from excessive GWG was higher in this study (OR = 1.98) than with IOM (OR = 1.69) (Table 3). In the normal weight group, the risk of MPTB associated with insufficient GWG was higher in this study (OR = 1.93 vs. 1.41). In the obese group. The IOM guidelines did not identify an association between excessive GWG and gestational age extension reduction.

Our findings are consistent with previous studies on twin pregnancies, indicating that inadequate and excessive GWG are associated with adverse perinatal risks [11,17,18]. Several Western studies confirm associations between abnormal GWG and LBW, PTB, SGA, and LGA in twin pregnancies [15,19,20]. For example, Lipworth et al. defined GWG below guideline recommendations as inadequate and found higher PTB and SGA with inadequate GWG, whereas excessive GWG increased the risk of hypertensive disorders of pregnancy15. Similarly, using a large U.S. database, Lin et al. reported inadequate GWG increased PTB and SGA, whereas excessive GWG increased LGA [8]. Although studies on GWG in Asian twin pregnancies are limited, available evidence shows similar trends and suggests the 2009 IOM guidelines may not be fully applicable to Asian populations. For example, Shimura et al. reported a lower optimal GWG range for Japanese women than IOM recommendations, with a median GWG of 13.6 kg in normal-BMI women, below the IOM lower limit of 16.8 kg [21]. Overall, both Western and Asian populations show that suboptimal GWG, either too low or too high, is associated with increased risks of adverse outcomes in twin pregnancies.

Our calculated optimal GWG ranges for Chinese women were generally lower than the IOM recommendations. In the normal BMI group, our suggested range was 14.80–21.46 kg, which is lower than the IOM’s 16.8–24.5 kg. This is more in line with the typically lower BMI observed among Chinese women. Asian women differ from Western women in terms of stature, body composition, fat distribution, and metabolic characteristics. Specifically, the median BMI in normal-weight Asian women is significantly lower than in Western populations (21.5 vs. 23.8 kg/m^2^) [22,23], and body fat tends to be more visceral [24]. Therefore, a lower upper limit for GWG may help reduce metabolic burden in this population.

Recent studies from Asian populations further support the need for adjusted GWG recommendations tailored to this demographic. A Korean cohort study found that women with twin pregnancies who gained less than the IOM recommendations had a significantly higher risk of PTB (aOR = 2.33), suggesting that guideline thresholds may need to be reduced to better suit Asian populations [25]. Japanese studies have also proposed that the IOM guidelines may overestimate GWG needs in Asian women, and lowering the upper limit could help reduce LGA risk [26]. For instance, Shimura et al. reported that the optimal median GWG for normal-BMI twin pregnancies in Japan was 13.6 kg—well below the IOM’s lower limit of 16.8 kg [21]. A recent Chinese study by Gao et al. (Scientific Reports, 2023) also proposed more conservative GWG ranges in a Southwest China twin cohort, with the optimal GWG range for normal-BMI women around 15–21.1 kg, again lower than IOM recommendations [13]. Considering both ethnic differences and local evidence, revising the GWG guidelines for Asian women is a rational and necessary step toward improving perinatal outcomes.

### 4.2. Biological plausibility

GWG likely acts through placental and metabolic pathways. Inadequate GWG reflects maternal undernutrition, limited plasma-volume expansion, and suboptimal placentation, impairing placental perfusion and fetal nutrient delivery. These conditions can activate fetal stress responses, such as hypothalamic–pituitary–adrenal axis activation, leading to growth restriction and preterm birth [27,28]. Conversely, excessive GWG indicates increased adiposity and insulin resistance, increasing risk of glucose intolerance and gestational diabetes [29]. Inflammation and oxidative stress–related endothelial dysfunction increase risk of preeclampsia and other hypertensive disorders of pregnancy [30]. Together, these changes compromise transplacental nutrient transfer and link to preterm birth and placental abruption. Insulin resistance and dysglycemia emerge by mid-pregnancy and are more pronounced in overweight or obese women, increasing gestational diabetes risk [31]. Overall, inadequate GWG signals limited reserves and insufficient blood-volume expansion, impairing placental function and fetal growth and raising SGA and preterm birth risk, whereas excessive GWG reflects metabolic overload, increasing maternal–fetal complications and affecting fetal growth and gestational length [32,33].

Given that fetal growth patterns in twin pregnancies differ from those in singleton pregnancies, the use of singleton-based growth standards may lead to overestimation of SGA and underestimation of LGA [16]. Reanalysis using twin-specific growth standards yielded more robust estimates, underscoring the methodological importance of applying population-appropriate references in twin research.

### 4.3. Strengths and limitations

Strengths include a large retrospective cohort of 3,109 twin pregnancies and the development of GWG reference ranges tailored for Chinese women. We systematically examined the associations between GWG and perinatal outcomes using threshold-curve models and multiple sensitivity analyses. Detailed maternal and pregnancy data were collected, and models were adjusted for numerous potential confounders. In this study, we used the 25th–75th percentile of gestational weight gain (GWG) as a reference range for the low-risk subgroup. While this approach is reasonable and consistent with similar studies (e.g. Lin et al.) [8], it is somewhat arbitrary and may not fully capture the full spectrum of ‘optimal’ GWG. Therefore, future research could explore alternative methods to more accurately define the optimal GWG range. One potential alternative is to use receiver operating characteristic (ROC) curves to determine GWG cutoffs that maximize sensitivity and specificity for adverse pregnancy outcomes. ROC curves provide a robust approach for identifying GWG thresholds that most effectively predict negative outcomes, thus improving the predictive accuracy of the model. This method could offer a more personalized approach for defining optimal GWG ranges in different subgroups. Limitations include the self-reported pre-pregnancy weight may introduce minor recall bias, potentially under- or overestimating BMI and GWG.; the possibility of residual confounding (e.g. lifestyle, psychological factors, clinical management); Since this study is a single-center investigation conducted in Northwest China, the findings may not be fully applicable to other regions, such as South China, where variations in diet and ethnicity may exist. Future multicenter studies are essential to validate these results across diverse populations.The relatively small number of obese women in the cohort may limit the precision of estimates for this subgroup. The study focused on short-term perinatal outcomes, and long-term follow-up data and nutritional/metabolic biomarkers were unavailable. Future multicenter and interventional studies are warranted to validate and extend these findings.

## 5. Conclusions

Based on a large cohort of twin pregnancies, this study proposes BMI-specific GWG recommendations, which are generally lower than those proposed by the IOM. Inadequate and excessive GWG both resulted in a higher risk of adverse perinatal outcomes.

## Supplementary Material

Supplementary Table.docx

## Data Availability

The data are available from the corresponding author upon reasonable request.

## Presentation

Not applicable/not previously presented.

## Clinical trial registration

Not applicable (retrospective observational study).
