# Supplementary material for: Gestational weight gain and perinatal outcomes in twin pregnancies: evidence-based insights from a Chinese population cohort
Source: Ann Med. 2026 Apr 27;58(1):2659391. doi: 10.1080/07853890.2026.2659391 (PMC13123048; doi:10.1080/07853890.2026.2659391)
Supplement: Supplementary Table.docx [file IANN_A_2659391_SM0902.docx]

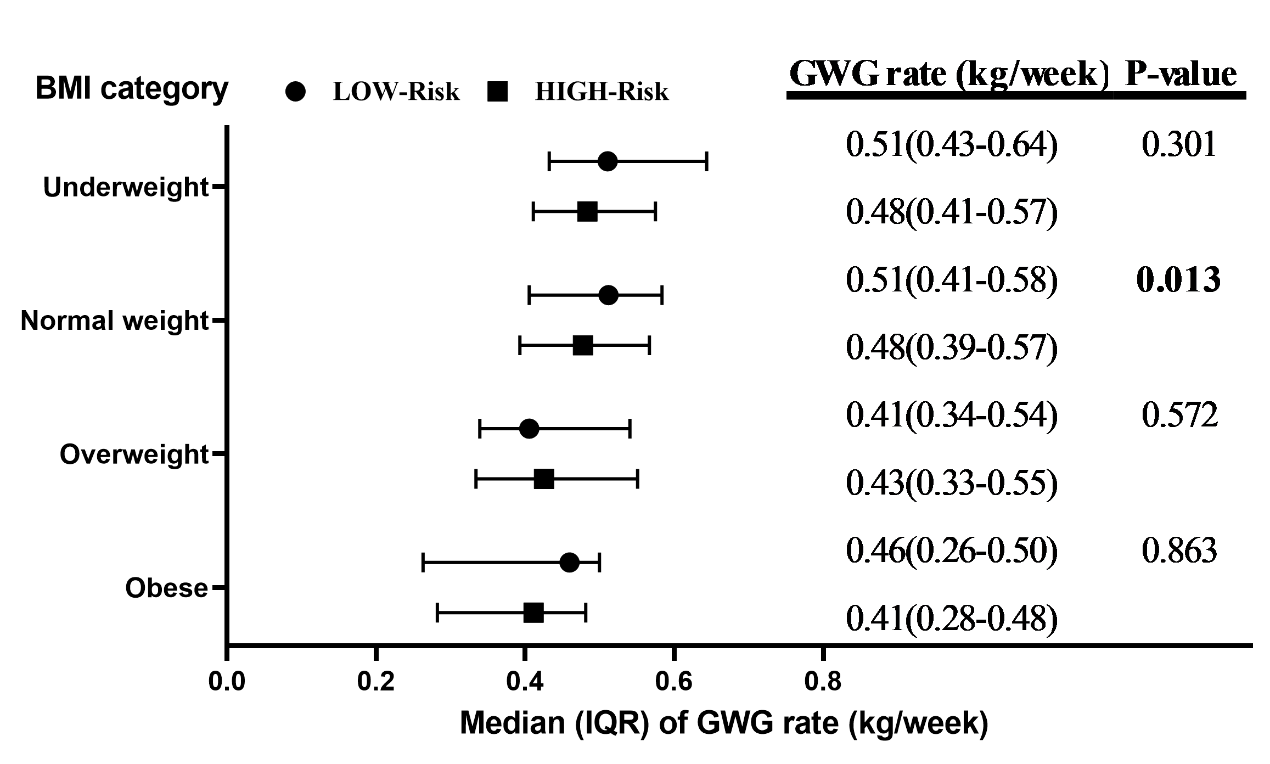
**Figure S1.** Distribution of GWG rates in low-risk and high-risk subgroups stratified according to the Chinese BMI classification standard.

Note: Median (interquartile range, IQR) of GWG rates (kg/wk) in low-risk and high-risk subgroups, stratified by Chinese BMI categories. The low-risk group was defined based on the IOM recommendations for gestational weight gain in twin pregnancies, including delivery of two live-born infants with birth weights ≥2500g, 5-minute Apgar scores ≥7, and gestational age at delivery >37 weeks. All other pregnancies were classified as high-risk.

**Table S1.** Our study(Chinese) recommendations for gestational weight gain in twin gestations

|  | | Our study recommended | | |
| --- | --- | --- | --- | --- |
| Pre-pregnancy BMI | Total weight gain at 37 wk in kg (lb) | |  | Weekly weight gain kg/wk (lb/wk) |
| Underweight (BMI<18.5 kg/m^2^) | 15.91–23.68(35.15–52.17) | |  | 0.43-0.64(0.95–1.41) |
| Normal weight (18.5−23.9 kg/m^2^) | 15.17–21.46(33.30–47.36) | |  | 0.41-0.58(0.90–1.28) |
| Overweight (24−27.9 kg/m^2^) | 12.58–19.98(27.75–44.03) | |  | 0.34-0.54(0.75–1.19) |
| Obese (≥28 kg/m^2^) | 9.62–18.50(21.09–40.70) | |  | 0.26-0.50(0.57–1.10) |

Note: The study-recommended GWG ranges are based on the interquartile range (25th–75th percentiles) of GWG in the low-risk group and stratified by Chinese BMI categories. lb is the abbreviation of pound. wk is short for week.

**Table S2.** Association of Gestational Weight Gain Categories Based on the IOM and Study Recommendations with Perinatal Outcomes Stratified by Chinese BMI classification standard

| BMI categories | Perinatal Outcomes | GWG rate  below IOM | GWG rate  above IOM | GWG rate  below our study | GWG rate  above our study |
| --- | --- | --- | --- | --- | --- |
|  |  | β/OR(95% CI) | β/OR(95% CI) | β/OR(95% CI) | β/OR(95% CI) |
| Normal weight | Cesarean delivery | 0.55 (0.28, 1.09) | 0.73 (0.26, 2.10) | 0.53 (0.26, 1.06) | 0.95 (0.40, 2.26) |
|  | Gestational age(week) | -0.29 (-0.45, -0.13) ^**^ | 0.05 (-0.19, 0.29) | -0.45 (-0.63, -0.27)^**^ | -0.04 (-0.24, 0.15) |
|  | PTB | 1.11 (0.84, 1.47) | 0.78 (0.52, 1.16) | 0.74 (0.55, 1.01) | 0.81 (0.58, 1.14) |
|  | MPTB | 1.44 (1.07, 1.93) ^*^ | 0.88 (0.55, 1.41) | 1.94 (1.42, 2.66) ^**^ | 1.20 (0.83, 1.72) |
|  | VPTB | 1.88 (1.16, 3.03) ^**^ | 1.09 (0.52, 2.30) | 2.54 (1.56, 4.15) ^**^ | 0.94 (0.50, 1.76) |
|  | Birth weight(g) | -112.04(-146.49,-77.60) ^**^ | 85.45(33.44,137.45) | -153.93 (-192.30, -115.57) ^**^ | 50.11 (7.41, 92.82) ^*^ |
|  | LBW | 1.59 (1.34, 1.88) ^**^ | 0.69 (0.54, 0.88) ^**^ | 1.97 (1.63, 2.38) ^**^ | 0.76 (0.63, 0.93) |
|  | VLBW | 1.61 (1.06, 2.44) ^*^ | 0.66 (0.32, 1.36) | 2.26 (1.44, 3.56) ^**^ | 1.02 (0.59, 1.77) |
|  | SGA | 1.17 (0.91, 1.49) | 0.76 (0.56, 1.03) | 1.17 (0.91, 1.49) | 0.76 (0.56, 1.03) |
|  | LGA | 0.45 (0.30, 0.66) ^**^ | 1.85 (1.40, 2.45) ^**^ | 0.45 (0.30, 0.66) ^**^ | 1.85 (1.40, 2.45) ^**^ |
|  | SVN | 0.9 (0.7, 1.3) | 0.9 (0.6, 1.3) | 0.9 (0.7, 1.3) | 0.9 (0.6, 1.3) |
|  | Preterm non-SGA | 0.81 (0.65, 1.02) | 1.13 (0.88, 1.45) | 0.81 (0.65, 1.02) | 1.13 (0.88, 1.45) |
|  | Term SGA | 2.78 (1.04, 7.43) ^*^ | 1.74 (0.54, 5.69) | 2.78 (1.04, 7.43) ^*^ | 1.74 (0.54, 5.69) |
|  | Preterm SGA | 1.05 (0.80, 1.38) | 0.69 (0.51, 0.95) ^*^ | 1.05 (0.80, 1.38) | 0.69 (0.51, 0.95) ^*^ |
|  | Apagar1≤7 | 1.20 (0.71, 2.02) | 0.90 (0.42, 1.95) | 1.68 (0.95, 2.99) | 1.32 (0.72, 2.41) |
|  | Apagar5≤7 | 0.54 (0.15, 1.99) | 1.82 (0.57, 5.77) | 1.83 (0.44, 7.60) | 4.26 (1.31, 13.80) ^*^ |
| Overweight | Cesarean delivery | 0.69 (0.26, 1.87) | 2.39 (0.26, 22.02) | 0.79 (0.27, 2.26) | 1.96 (0.50, 7.66) |
|  | Gestational age(week) | -0.18 (-0.51, 0.14) | 0.17 (-0.37, 0.71) | -0.28 (-0.65, 0.10) | 0.39 (0.02, 0.76) ^*^ |
|  | PTB | 1.01 (0.59, 1.75) | 0.63 (0.28, 1.45) | 1.21 (0.65, 2.27) | 0.89 (0.49, 1.63) |
|  | MPTB | 1.21 (0.74, 1.98) | 1.00 (0.43, 2.32) | 1.44 (0.84, 2.45) | 0.57 (0.31, 1.05) |
|  | VPTB | 1.12 (0.57, 2.19) | 0.71 (0.20, 2.54) | 1.22 (0.58, 2.55) | 0.44 (0.18, 1.06) |
|  | Birth weight(g) | -149.38 (-221.95, -76.80) ^**^ | 106.59 (-5.91, 219.09) | -130.05 (-219.73, -40.38) ^**^ | 157.67 (82.87, 232.46) ^**^ |
|  | LBW | 1.69 (1.25, 2.29) ^**^ | 0.55 (0.34, 0.90) ^*^ | 1.67 (1.17, 2.40) ^**^ | 0.55 (0.39, 0.78) ^**^ |
|  | VLBW | 2.06 (1.05, 4.02) ^*^ | 0.60 (0.17, 2.09) | 1.93 (0.93, 3.99) | 0.49 (0.21, 1.15) |
|  | SGA | 1.65 (1.03, 2.66) ^*^ | 0.89 (0.50, 1.58) | 1.30 (0.80, 2.09) | 0.79 (0.47, 1.34) |
|  | LGA | 0.51 (0.31, 0.82) ^*^ | 1.73 (1.10, 2.71) ^**^ | 0.67 (0.40, 1.11) | 1.86 (1.22, 2.86) ^**^ |
|  | SVN | 0.9 (0.5, 1.7) | 0.8 (0.4, 1.6) | 1.1 (0.6, 2.3) | 1.0 (0.5, 1.9) |
|  | Preterm non-SGA | 0.65 (0.43, 0.97) ^*^ | 0.97 (0.59, 1.60) | 0.85 (0.55, 1.31) | 1.25 (0.79, 1.98) |
|  | Term SGA | 0.54 (0.09, 3.07) | 0.57 (0.11, 3.13) | / | 0.62 (0.12, 3.24) |
|  | Preterm SGA | 2.02 (1.24, 3.30) ^**^ | 0.93 (0.50, 1.75) | 1.59 (0.97, 2.61) | 0.71 (0.40, 1.25) |
|  | Apagar1≤7 | 2.87 (1.32, 6.25) ^**^ | 1.49 (0.44, 5.11) | 2.44 (1.10, 5.41) ^*^ | 0.68 (0.26, 1.77) |
|  | Apagar5≤7 | 37.22 (8.04, 172.23) ^**^ | 84.27 (8.91, 797.47) ^**^ | 10.35 (1.38, 77.74) ^*^ | 4.19 (0.48, 36.62) |
| Obese | Cesarean delivery | 0.10 (0.00, 4.92) | / | 9.57 (0.10, 899.65) | 21.68 (0.02, 23469.91) |
|  | Gestational age(week) | -0.92 (-1.65, -0.20) ^*^ | 0.41 (-0.72, 1.54) | -0.70 (-1.52, 0.13) | 0.91 (0.11, 1.71) ^*^ |
|  | PTB | 1.05 (0.36, 3.06) | 1.93 (0.19, 19.32) | 0.94 (0.30, 2.98) | 0.72 (0.21, 2.55) |
|  | MPTB | 2.94 (1.19, 7.24) ^*^ | 0.57 (0.12, 2.80) | 2.05 (0.76, 5.50) | 0.25 (0.08, 0.84) ^*^ |
|  | VPTB | 3.30 (0.95, 11.51) | 0.61 (0.05, 7.26) | 1.69 (0.46, 6.26) | 0.24 (0.04, 1.47) |
|  | Birth weight(g) | -239.80 (-405.77, -73.82) ^**^ | 137.31 (-73.43, 348.05) | -148.40 (-319.61, 22.82) | 393.12 (222.30, 563.93) ^**^ |
|  | LBW | 2.14 (1.15, 3.99) ^*^ | 0.70 (0.27, 1.83) | 1.65 (0.78, 3.48) | 0.26 (0.13, 0.55) ^**^ |
|  | VLBW | 2.89 (0.84, 9.99) | 0.31 (0.03, 3.36) | 1.37 (0.37, 5.11) | 0.31 (0.09, 1.07) |
|  | SGA | 1.17 (0.48, 2.83) | 0.51 (0.19, 1.35) | 1.20 (0.46, 3.14) | 0.41 (0.16, 0.98) ^*^ |
|  | LGA | 1.60 (0.67, 3.83) | 5.51 (2.26, 13.41) ^**^ | 1.08 (0.42, 2.78) | 4.45 (1.78, 11.10) ^**^ |
|  | SVN | 1.0 (0.2, 4.4) | 0.7 (0.1, 4.0) | 0.6 (0.1, 2.4) | 0.7 (0.1, 3.7) |
|  | Preterm non-SGA | 1.02 (0.45, 2.29) | 1.27 (0.52, 3.12) | 0.79 (0.34, 1.81) | 1.44 (0.61, 3.38) |
|  | Term SGA | / | 0.90 (0.16, 5.22) | / | / |
|  | Preterm SGA | 1.28 (0.51, 3.23) | 0.49 (0.17, 1.41) | 0.74 (0.29, 1.89) | 0.32 (0.12, 0.86) * |
|  | Apagar1≤7 | 16.08 (1.16, 223.54) ^*^ | / | 13.94 (1.98, 98.35) ^**^ | / |
|  | Apagar5≤7 | / | / | 4.93 (4.93, 4.93) ^**^ | / |

Note: The study-recommended GWG ranges are based on the interquartile range (25th–75th percentiles) of GWG in the low-risk group and stratified by Chinese BMI categories.SD, standard deviation; PTB, preterm birth; MPTB, moderate preterm birth; VPTB, very preterm birth; LBW: low birth weight; VLBW, very low birth weight; SGA, small for gestational age; LGA, large for gestational age; SVN, small vulnerable newborns. Adjust model adjust for: maternal age, educational status, gravidity, parity, pregnancy loss history, PCOS (Polycystic Ovary Syndrome), pre-pregnancy diabetes, GDM (gestational diabetes mellitus), pre-pregnancy hypertension, GHDs (gestational hypertensive disorders), Past cesarean delivery, Methods of conception, Chorionicity, BMI (body mass index). **P*＜.05, ** *P*＜.01.
